# Motives for competitive sports participation in masters track and field athletes: Impact of sociodemographic factors and competitive background

**DOI:** 10.1371/journal.pone.0275900

**Published:** 2022-11-02

**Authors:** Titta K. Kontro, Dmitriy Bondarev, Kukka-Maaria Pyykönen, Mikaela von Bonsdorff, Lauri Laakso, Harri Suominen, Marko T. Korhonen

**Affiliations:** 1 Faculty of Sport and Health Sciences, University of Jyväskylä, Jyväskylä, Finland; 2 Gerontology Research Center, Faculty of Sport and Health Sciences, University of Jyväskylä, Jyväskylä, Finland; Université de Lille: Universite de Lille, FRANCE

## Abstract

**Objectives:**

Masters athletes due to their lifelong engagement in sport represent a unique group to study motivation for regular physical activity, but there is less scientific data on the sport motives in masters athletes. Therefore, the aim of this study was to evaluate the association of age, sex, education, nationality, competitive background and training amount with sport motives of masters track and field athletes.

**Methods:**

811 (254 women) athletes aged 35–89 years participated in European Veterans Athletics Championships in the year of 2000. Sport motives were assessed with a questionnaire and categorized into1) well-being, 2) competitive and performance 3) health and fitness motives.

**Results:**

Men considered health and fitness motives more important than women (p = 0.022). Over 65-year-old athletes considered health and fitness motives more important than the youngest, 35-49-year age group (p<0.001). Education was not associated with sport motives, while motives varied significantly among different nationalities. Athletes from Nordic Countries considered well-being motives more important than Southern Europeans (p<0.001) or Western Europeans (p<0.05). Athletes from Nordic Countries (p = 0.003), Eastern Europe (p<0.001) and Central Europe (p<0.001) found health and fitness motives more meaningful than athletes from the British Isles. Furthermore, athletes with competitive background before the age of 35 and training amount more than 7.5 h a week found competitive and performance motives more important than athletes without a competitive history (p = 0.002).

**Conclusions:**

These results suggest that age, sex, nationality and former athleticism are associated with sports motives among international level masters track and field athletes. It seems that while for masters athletes with competitive background performance motives dominate, for older adults, particularly for males, health and fitness motives are more important. In addition, when designing the exercise interventions for older adults, different sport motives across countries should be considered.

## 1. Introduction

There is a widespread public health policy and scientific consensus that regular physical activity (PA) is an essential component of successful ageing and that PA reduces the risk of developing several age- and lifestyle-related diseases [1–3]. However, sedentary behavior is very common in the general population and it is still a major challenge to get especially older adults exercise and maintain their participation in exercise programs [4, 5]. Currently only about 10% of adults in general population meet the minimum recommended level of PA [5].

Studies have shown that middle-aged and older “veteran/masters” athletes (aged 35 and over) have substantially better physical performance and they are generally healthier than their sedentary peers [6, 7]. Therefore, masters athletes provide a unique research cohort to understand the role of regular vigorous exercise in optimizing physical potential and health with advancing age [6, 8].

Besides issues related to physical function and health, the study of masters athletes may also provide valuable insight into the motives associated with continued participation in sports [9–11]. A few studies have found that age may influence motives for sport participation so that extrinsic motives such as health may be more important for older than for younger athletes [8, 12]. It has also been reported that in male masters athletes sport motives are often associated with competing and performance, while in women health and social motives are more important [8]. However, findings from one investigation suggest that sex differences in sports motives may become less evident in later life [13]. Some studies in non-athletic people have also reported that higher education correlates positively with the continuation of the sport participation and sport motivation [11, 14].

Research about motives for athletic training and competitions has long focused primarily on young athletes, while studies available for masters athletes are few and limited in scope. Little is known about whether the motives for participating in masters sports vary with age, sex and education and to the best of our knowledge no data are available on the potential influences of competitive background and nationality. Previous studies have found that the age-related decline track and field performance in masters athletes is dependent on sex and event [15–17]. However, it has been also observed that in both female and male masters athletes the improvements in performances have been relatively small over the past few decades [15–17]. Investigating sport motivation in aging athletes may get new insights into the reasons for engaging in and maintenance of physically active lifestyle, and a topic that is of great importance also from a public health perspective [18].

This topic can be of importance also from public health perspective because lifelong engagement in relatively vigorous exercise is known to be effective strategy for promoting several physiological characteristics and health into advanced age. The aim of the present study, therefore, was to carry out a comprehensive examination of the factors that may be linked to participation motives in competitive sports in international level masters athletes. The specific aims and *hypotheses* were as follows:

1. Aim#1: to evaluate age-related differences in participation motives in competitive sports;
  *Hypothesis#1*: *Health motives are more important for older than younger athletes* [19].
2. Aim#2: to compare sports motives between men and women;
  *Hypothesis#2*: *Male motives are associated with competing and performance*, *and female motives are tended to associate with health and social factors* [18].
3. Aim#3: to determine whether sports motives differ between athletes with competition experience before age 35 versus those who engaged in competitions for the first time in masters athletics;
  *Hypothesis#3*: *Those participants who have experience in competitive athletics in youth find competitive and performance motives more important than those without earlier life competition background* [20].
4. Aim#4: to examine if current training amount associates with sports motives;
  *Hypothesis#4*: *The current training is linked to sport motivation*. *Previous study found association years of training and sport motivation* [21].
5. Aim#5: to evaluate the relation of education level to sport motives;
  *Hypothesis#5*: *The higher level of education correlates positively with the continuation of sport participation and sport motivation* [11, 14].
6. Aim#6: to determine whether there are differences in sports motives among athletes of different nationalities.
  *Hypothesis#6*: *Athletes from Nordic Europe find competitive motives more important than athletes from Eastern and Western Europe* [22].

## 2. Methods

### 2.1 Participants

This study was a part of a larger research project carried out during the XII European Veterans Athletics Championships held in Jyväskylä, Finland (July 2000), in which 2900 athletes from 40 countries were registered. The athletes were invited by personal letter to participate in the questionnaire study. A total of 811 questionnaires were returned of which 254 were from women and 557 were from men with an age range of 35–89 years (M = 56.2, SD = 11.5) (Table 1). The respondents were from 24 European countries. All participants provided their written informed consents. The study was approved by the local organizing committee and the research protocol was approved by the Ethics Committee of the University of Jyväskylä.

**Table 1:** Descriptive information of the participants.

Variables	Total n	Mean (SD) or n (%)
**Age, years**	**806**	**56.1 (11.4)**
**Sex**	**811**	
**female**		**254 (31)**
**male**		**566 (69)**
**Marital status**	**806**	
Single		**75 (10)**
Married or cohabiting		**641 (79)**
Divorced, separated or widowed		**90 (11)**
**Country group**	**811**	
Northern Europe		**259 (32)**
Western Europe		**59 (7)**
Central Europe		**228 (28)**
Eastern Europe		**75 (9)**
Southern Europe		**85 (10)**
British and IrishIsles		**105 (13)**
**Education**	**811**	
Primary		**156 (19)**
Secondary		**318 (39)**
Tertiary		**330 (40)**
**Competitive background, participation in competitions under age 35 (yes)**	**809**	**655 (81)**
**Current training**, hours per week	**702**	
Less than 5 h		
From 5 to 7.5 h		**195 (28)**
More than 7.5 h		**251 (36)**
		**256 (36)**
**Years of training**	**636**	**11.3 (8.7)**

### 2.2 Questionnaire

The questionnaire consisted of 34 items and included information on country, age, sex, education, competitive background, current training, injuries and sports motives. The questionnaire was available in eight different languages (English, Finnish, French, German, Italian, Russian, Spanish and Swedish) and administered as pen and paper format. *Level of education* was assessed using a single question, and categorized as primary, secondary and tertiary (applied science degree, bachelor’s degree, nurse training, master’s degree, and PhD) levels. *Competitive background* was assessed as a dichotomous variable, participants had to indicate as yes or no whether they participated in competitive sport before 35 years of age. *Current training* was calculated as the average summer and winter training hours per week during the past 12 months. Respondents were divided into three categories: less than 5 hours, from 5 to 7.5 hours and more than 7.5 hours per week.

*Sports motives* were assessed with the 17-item questionnaire. The participants had to answer a question “What does veteran sport mean to you personally?” on a 3-point scale: 1 (not at all important); 2 (slightly important); 3 (very important). The response items were: It prevents illnesses; It is a good way to lose or control my weight; It has become a habit; It is a good way to relax and freshen up; I enjoy competing; I enjoy the physical exertion; I enjoy the feeling of control of my body; I meet nice people when doing sports; It is essential for staying healthy; I enjoy winning; Training increases my sexual vigor; It is a good way to spend time out in the nature; I freshen up and feel joy from training; I get an increased feeling of well-being after exercising; It keeps me youngish; I get to be alone when exercising; I can measure my performance by following my results.

### 2.3 Statistical analysis

Athletes were grouped into six country groups: Northern Europe (participants from Denmark, Finland, Norway and Sweden); Western Europe (Belgium, France and the Netherlands); Central Europe (Austria, Germany and Switzerland); Eastern Europe (Estonia, Hungary, Poland, Latvia, Lithuania, Russia, Slovakia, Czech Republic and Ukraine); Southern Europe (Greece, Italy and Spain); British and Irish Isles (Great Britain and Ireland).

Principal components analysis (PCA) with oblique rotation was used to group the responses of the sport motives questions into meaningful categories with no a priory hypothesis. According to the recommendations of Tabachnick et al. (2007) [23], if correlation coefficients between items are generally greater than 0.32 then oblique rotation is warranted. In our data, correlation coefficients ranged from 0.13 to 0.62 thus justifying the use of oblique rotation. This analysis yielded 4 factors with Eigenvalues of 1.0 or greater. A minimal loading of 0.40 was used as a criterion value in the interpretation of individual factors [23]. As several items loaded on different factors, based on theoretical grounds and interpretability, we re-run the oblique rotation with a specification of 3factors. Three factors solutions accounted for 43.1% of the variance of the variables. Finally, based on the items that loaded on factors, the factor names and percentage of variance accounted for were as follows a) well-being motives, 25.6%, b) competition and performance motives 10.2%, and c) health and fitness motives, 7.3%. The factor of well-being consisted of 7 items, with the Cronbach’s alpha 0.69. The factor of competition and performance consisted of 5 items, with the Cronbach’s alpha 0.70. The factor of health and fitness consisted of 5 items, with the Cronbach’s alpha 0.68.

To test differences in sport motives scores across the studied variables, analysis of variance (ANOVA) was used with the post hoc Tukey test. P-values <0.05 were considered statistically significant. Statistical analyses were performed using SPSS statistical software (IBM SPSS Statistics for Windows, Version 26.0. Armonk, NY: IBM Corp).

## 3. Results

The majority of participants came from Northern Europe (32%), followed by Central Europe (28%), British and Irish Isles (13%), Southern Europe (10%), Eastern Europe (9%), and Western Europe (7%) (Table 1). Almost an equal number of all participants reported to have tertiary and secondary degree, and every fifth had primary degree of education. Four in five participants had competition experience before 35 years of age. The athletes trained an average of 6.7 (SD 3.2) hours per week (range 0.1–25.0) in summer and of 6.5 (SD 3.3) hours per week (range 0.2–22.5) in winter (not shown in the Table). Twenty-eight percent of the participants trained less than 5 hours per week, 36% from 5 to 7.5 hours per week and 36% more than 7.5 hours per week during the past year (Table 1).

Table 2 shows a variation in sport motives across age, sex, education, country groups, competitive background and training amount. The athletes over 65 years of age considered health and fitness motives more important than the youngest (35–49 years) age group (p<0.001). Men considered health and fitness motives more important than women (p = 0.022). However, no differences were seen between different levels of education (p>0.05). Athletes from Nordic Countries (p = 0.003), Eastern Europe (p<0.001) and Central Europe (p<0.001) found health and fitness motives more meaningful than athletes from the British and Irish Isles. Athletes from Nordic Countries considered well-being motives more important than Southern European (p<0.001) and Western European (p<0.05) athletes. Additionally, both Eastern (p = 0.003) and Western European(p<0.001)athletes found well-being motives more important than Southern European athletes. Athletes with competition experience before age 35 found competitive and performance motives more important than athletes who had begun to compete after age 35 (p = 0.002). Correspondingly, athletes currently training more than 7.5 hours per week found competitive and performance motives more important than athletes training less than 5 hours per week (p = 0.005).

**Table 2:** Variation in sport motives across age, sex, education, country, competitive background and training amount.

	Sport motives					
	Well-being		Competition and performance		Health and fitness	
	*n*	Mean (SD)	*n*	Mean (SD)	*n*	Mean (SD)
**Age groups**						
49 and younger,	234	2.39 (0.38)	244	2.41(0.47)	234	2.14 (0.47)
50–59,	196	2.42 (0.37)	205	2.48 (0.40)	195	2.23 (0.44)
60 and older,	259	2.45 (0.38)	277	2.42 (0.43)	259	2.31 (0.43)
ANOVA *p*-value for difference		0.205		0.145		<**0.001**
**Sex**						
Male	477	2.40 (0.38)	505	2.44 (0.44)	480	2.26 (0.45)
Female	215	2.45 (0.37)	225	2.42 (0.44)	205	2.17 (0.46)
ANOVA *p*-value for difference		0.120		0.575		**0.022**
**Education**						
Primary	133	2.44 (0.39)	143	2.47 (0.45)	128	2.28 (0.47)
Secondary	262	2.45 (0.39)	280	2.46 (0.41)	265	2.23 (0.46)
Tertiary	293	2.39 (0.36)	303	2.40 (0.45)	287	2.21 (0.44)
ANOVA *p*-value for difference		0.168		0.138		0.378
**Country**						
Northern Europe	220	2.51 (0.35)	232	2.39 (0.45)	219	2.24 (0.43)
Western Europe	51	2.34 (0.48)*	55	2.42 (0.49)	48	2.11 (0.54)
Eastern Europe	52	2.51 (0.34)	55	2.43 (0.42)	50	2.37 (0.44)
Southern Europe	69	2.27 (0.38)*	72	2.33 (0.41)	66	2.19 (0.48)
Central Europe	199	2.50 (0.34)	213	2.51 (0.43)	203	2.33 (0.43)
British and Irish isles	101	2.16 (0.38)*	103	2.49 (0.37)	99	2.04 (0.43)
ANOVA *p*-value for difference		<**0.001**		**0.16**		<**0.001**
Significant post hoc differences	NE>WE, SE		NE<CE,		NE>Br&I,	
	WE<CE		SE<CE		WE<CE,	
	EE>SE				EE>Br&I,	
	SE<CE				CE>Br&I	
	CE>Br&I					
**Competitive background (< 35 y)**						
Yes	560	2.41 (0.38)	590	2.46 (0.42)	559	2.22 (0.45)
No	130	2.44 (0.36)	138	2.33 (0.48)		126 (2.30)
ANOVA *p*-value for difference		0.518		**0.002**		0.066
**Current training**						
Less than 5 h per week	168	2.37 (0.38)	176	2.36 (0.47)	163	2.21 (0.46)
5–7.5 h per week	220	2.42 (0.41)	232	2.44 (0.45)	219	2.24 (0.47)
More than 7.5 h per week	221	2.44 (0.35)	231	2.50 (0.38)	221	2.23 (0.44)
ANOVA *p*-value for differences		0.224		**0.010**		0.815
Significant post hoc differences				Less than 5h< 7.5h		

NE—Northern Europe, WE—West Europe, SE—South Europe, EE—East Europe, CE—South Europe, Br&I—British and Irish Isles

## 4. Discussion

We found that older athletes considered health and fitness motives more important than the athletes in the youngest age group. Men considered health and fitness motives more important than women. Athletes who had competitive experience in earlier life (<35 years) found competitive and performance motives more important than athletes without a background in competitive athletics. Athletes who trained on average less than 5 hours per week reported lower competition and performance motives than those who trained more than 7.5 hours per week. There were also differences in sport motivation among athletes of different country groups, whereas level of education was not associated with sport motives. Therefore, based on our main findings we can confirm four of our *hypotheses* (*#1* (age), *#2* (competitive background), *#4* (current training), #6 (nationality)), but not *hypotheses #2* (sex) and *#5* (education).

During the past three decades sport motivation among young elite athletes has been widely studied, but the sport motivation among competitive masters athletes remains minimally researched [8, 18]. The understanding of motivation to continue systematic training and competing as masters athlete is difficult because of the complex set motivational factors that may differ with age, sex, education and culture [8]. Our study addressed these issues and yielded a number of new and complementary results to previously known literature. We found that over 65-year-old athletes considered health and fitness motives more important than the youngest age group. In line with our results, a study with marathon runners has shown that health motives are more important for older than younger athletes [19]. One explanation for this finding could be that older people may consider sport participation as a mean to lead to healthier life [19]. However, giving the cross-sectional nature of existing studies on the link between age and sport motives, it is unclear whether sport motives change during one’s life, or the observed differences represent cohort variations and influence of other factors. Future studies need to use longitudinal data to verify aging-related changes in sports motivation.

Sex differences in participation motives for competitive masters sports are also widely recognized; male motives are often associated with competing and performance, and female motives tend to associate with health and social factors [18]. This view is partly inconsistent with our findings that men estimated health and fitness motives more important than women. It has also been shown that female masters athletes assign less importance to competition and extrinsic rewards [24]. In our study we did not observe sex difference in competition and performance motives. A study with athletes aged 45–80 years has shown that sex differences in sport motives disappear in later life [13]. Sex-related stereotype may prevent women’s active participation in competitive sports, particularly at later adult years [25]. Thus, it could be that female masters athletes may share similar sport motives with men to compensate sex-related stereotype. Longitudinal data could be used to examine changes in sex-related differences in sport motives during lifespan in the future.

Cultural/country differences have been found to be associated with sport motives among young athletes [26]. For example, intrinsic motivation was higher among young athletes from USA compared to athletes from Korea [27]. As far as we are aware, our study is the first to compare participation motives for competitive sports in masters athletes of different countries. Our study extends previous research conducted predominantly with young athletes to masters athletes and suggests that the athletes from Nordic Countries considered well-being motives more important than Southern Europeans or Western Europeans. Furthermore, athletes from Nordic Countries, Eastern Europe and Central Europe found health and fitness motives more meaningful than athletes from the British and Irish Isles. It was also found that among Central European athletes, health and fitness motives were considered more important than among Western European athletes. Recently, it has been shown that cultural differences affect the way that sport values are prioritized to achieve performance related goals in competitive sport [28]. Thus, country-specific issues may occur when considering motivational factors to engage older adults into regular exercise. Future studies may wish to identify specific sets of values that may be related to engagement in sport in a country-specific context.

It has also been reported that higher education correlates positively with the continuation of sport participation and sport motivation in older non-athletes [11, 14]. However, in the present study no differences in sport motives were seen between different levels of education. In our study, the majority of participants reported having a tertiary degree of education meaning that the lack of association between education and sport motives could be due to a relatively homogeneous group of well-educated participants. Higher education could be associated with higher income that makes possible to travel abroad to compete, this partially explains homogeneity of the study population. Future studies with more heterogeneous groups are needed to investigate how education is associated with sport motives at different levels of education at different ages.

Finally, we observed that athletes who had engaged in competitive athletics when they were young (<35 years) and those who trained more than 7.5 hours per week found competitive and performance motives more important than athletes without a competitive history in younger age and those who trained less than 5 hours a week, respectively. In a study on Spanish masters track and field athletes, it has been shown that the amount of training per week is not linked to motivational profiles, while years of training was associated with intrinsic motives [21]. Another study showed that performance or personal achievement motivation was also a dominant factor for marathon runners [29]. It seems that engagement in competitive sport for older adults may require a different set of motives than involvement in health-related PA. Future studies need to examine specific sets of values that may be related to engagement in sport at different levels of PA and competition.

It is well known that lack of regular PA in the general population contributes to several age-related diseases, functional decline which may subsequently prevent autonomous daily living in older age [1, 2, 30]. However, motivating these people to maintain physically active lifestyle or start exercising is still a major challenge [4, 5]. Based on the present results, it could be reasonable to suggest for such exercise programs to account health and fitness motives for adults previously not involved in active sport, particularly for men, or to incorporate a personal achievement motivational component for those who have more experience with sports. In addition, when designing the exercise interventions for older adults, different sport motives across countries should be considered. Future research is called for to explore country-specific motives for preparing and competing in sports to support evidence-based intervention for exercise participation.

The strength of the current study is that it was conducted with a relatively large and internationally representative group of masters athletes. Another major strength was that the questionnaire was translated into eight different languages (English, Finnish, French, German, Italian, Russian, Spanish and Swedish) that enabled to study for the first time the association of nationality (culture) on motivational factors in aging athletes. Our study had some limitations that should be mentioned. Although the data was collected years ago, the sport motivation is most likely a characteristic that is not affected by that time, and thus the results are still relevant. Furthermore, to the best of our knowledge no study has investigated the possible differences in sport motives across cohorts collected at various years. Even though the number of athletes that participated in the survey was relatively high, we cannot exclude self-selection bias when athletes with certain characteristics (e.g. higher education) would have participated in the study. Furthermore, self-reported data on amount of training might be susceptible to errors. However, athletes usually control their training patterns carefully using different means (e.g. exercise diaries), thus we may assume that over-reporting of exercise which is common in a general population is lower in athletic population [31].

## 5. Conclusions

In conclusion, the findings of this study indicated that age, sex, nationality and former athleticism are associated with sports motives among international level masters track and field athletes. It seems that while for masters athletes with a competitive background and greater amount of exercise performance motives dominate, for older adults, particularly for men, health and fitness motives are more important. In addition, motives vary significantly among different nationalities while education does not associate with sport motives among relatively well-educated masters athletes.
